# Efficacy Evaluation of Different Copper-Based Bactericides Against Citrus Canker and Candidate Role of Citrus *COPT* Genes in Response to *Xcc*

**DOI:** 10.3390/plants15213453

**Published:** 2026-11-05

**Authors:** Rongchun Ye, Jianming Luo, Ziqing Sun, Xinjie Long, Hongyan Fu, Naying Gao, Chenxing Hao, Ling Sheng, Yunlin Cao, Youhan Kong, Xianfeng Ma, Yan Jin, Jian Han

**Affiliations:** 1 Hunan Horticultural Research Institute, Hunan Academy of Agricultural Sciences, Changsha 410125, China; 2 Engineering Research Center for Horticultural Crop Germplasm Creation and New Variety Breeding, Ministry of Education, Hunan Agricultural University, Changsha 410128, China; 3 Yuelushan Laboratory, Changsha 410128, China; 4 National Center for Citrus Improvement, College of Horticulture, Hunan Agricultural University, Changsha 410128, China; 5 Key Laboratory of South China Agricultural Plant Molecular Analysis and Genetic Improvement and Guangdong Provincial Key Laboratory of Applied Botany, South China Botanical Garden, Chinese Academy of Sciences, Guangzhou 510650, China

**Keywords:** citrus canker, *Xanthomonas citri* subsp. *citri*, thiosen copper, copper-based bactericides, *COPT4*, copper homeostasis

## Abstract

Citrus canker, caused by *Xanthomonas citri subsp. citri* (*Xcc*), is commonly managed with copper-based formulations, yet the performance of individual products may differ between laboratory and plant assays. We compared 16 commercial copper-containing formulations in plate-diffusion and root-drench assays, and inhibition zones varied among products. Thiosen Copper, however, produced no visible inhibition zone on agar but suppressed *Xcc* growth after root application. In sweet orange, this suppression appeared earlier than with Bordeaux Mixture at the same nominal total Cu concentrations. Thiosen Copper raised the leaf chlorophyll index without a significant increase in total leaf Cu, whereas Bordeaux Mixture led to greater leaf Cu accumulation. In separate experiments with *Poncirus trifoliata*, the two formulations produced different growth and tissue Cu responses, and root treatment altered *PtCOPT* transcript levels. Transient overexpression of *CsCOPT4* in sweet orange leaves was accompanied by lower *Xcc* populations. These results reveal formulation-specific responses and nominate *CsCOPT4* as a candidate for further study.

## 1. Introduction

Citrus canker, caused by *Xanthomonas citri* subsp. *citri* (*Xcc*), damages leaves, shoots, and fruit, and can cause premature abscission and reduced fruit quality [1,2]. Copper-based sprays remain an important management option, although their effectiveness differs among formulations [3]. Genetic determinants of copper resistance have been characterized in *Xcc* [4], and prolonged copper use raises concerns about soil accumulation and harm to non-target organisms [5,6]. Under unfavorable conditions, citrus tissues can also suffer copper injury [7,8], and rootstocks differ in their physiological responses to excess Cu [9]. In orchards, disease pressure is further shaped by rainfall, wind, and local shelter [10]. Alternative approaches to suppressing *Xcc* have also been explored [11], but copper formulations remain of practical interest. Evaluating individual products and application routes therefore requires attention to both antibacterial activity and plant performance.

Copper formulations deposited on plant surfaces can inhibit bacterial growth as copper is released from the deposits [12]. Copper also plays roles in plant defense beyond its direct antimicrobial activity. In *Arabidopsis*, Cu^2+^-responsive signaling induces the ethylene-biosynthetic gene *ACS8*, and later work identified a CPK3–bHLH107–HY5 regulatory module involved in this response [13,14]. A pathogen-induced nuclear copper chaperone has likewise been implicated in *PR1* expression and resistance to *Pseudomonas syringae* [15]. These findings link copper homeostasis to immunity and raise the question of whether commercial copper formulations elicit similar responses in citrus. Copper allocation in plants involves several transporter families, including *COPTs*, heavy metal ATPases (HMAs), and yellow stripe-like proteins (YSLs) [16,17].

Copper transporters (*COPTs*) are conserved proteins that contribute to Cu uptake and intracellular distribution [18]. Plant *COPTs* generally contain three or four transmembrane helices and conserved motifs implicated in copper transport [19]. In *Arabidopsis*, *COPT1* is involved in root Cu acquisition [20], *COPT5* contributes to intracellular Cu remobilization [21], and *COPT6* influences Cu distribution under deficiency [22]. A connection to disease susceptibility has also been reported in rice, where *COPT1* and *COPT5* interact with the susceptibility protein *XA13* during infection by *Xanthomonas oryzae* pv. *oryzae* [23]. It remains unknown whether citrus *COPT* genes respond differently to commercial copper formulations and whether individual family members influence *Xcc* growth in leaves.

An earlier study examined the control of citrus canker by Thiosen Copper [24]. Here, we first screened 16 commercial copper-containing formulations in plate-diffusion and root-drench assays. We then examined Thiosen Copper in more detail, because it produced no visible inhibition zone on agar yet reduced *Xcc* growth after root application. Bordeaux Mixture, a conventional copper formulation, served as the comparator for physiological traits and tissue Cu concentrations. Finally, we measured the transcript responses of *PtCOPT* genes in *P. trifoliata* and transiently overexpressed the six corresponding *CsCOPT* genes in sweet orange leaves to test whether they affect *Xcc* growth.

## 2. Results

### 2.1. Plate-Diffusion Screening of Commercial Copper-Containing Formulations Against Xcc

**Figure 1:**
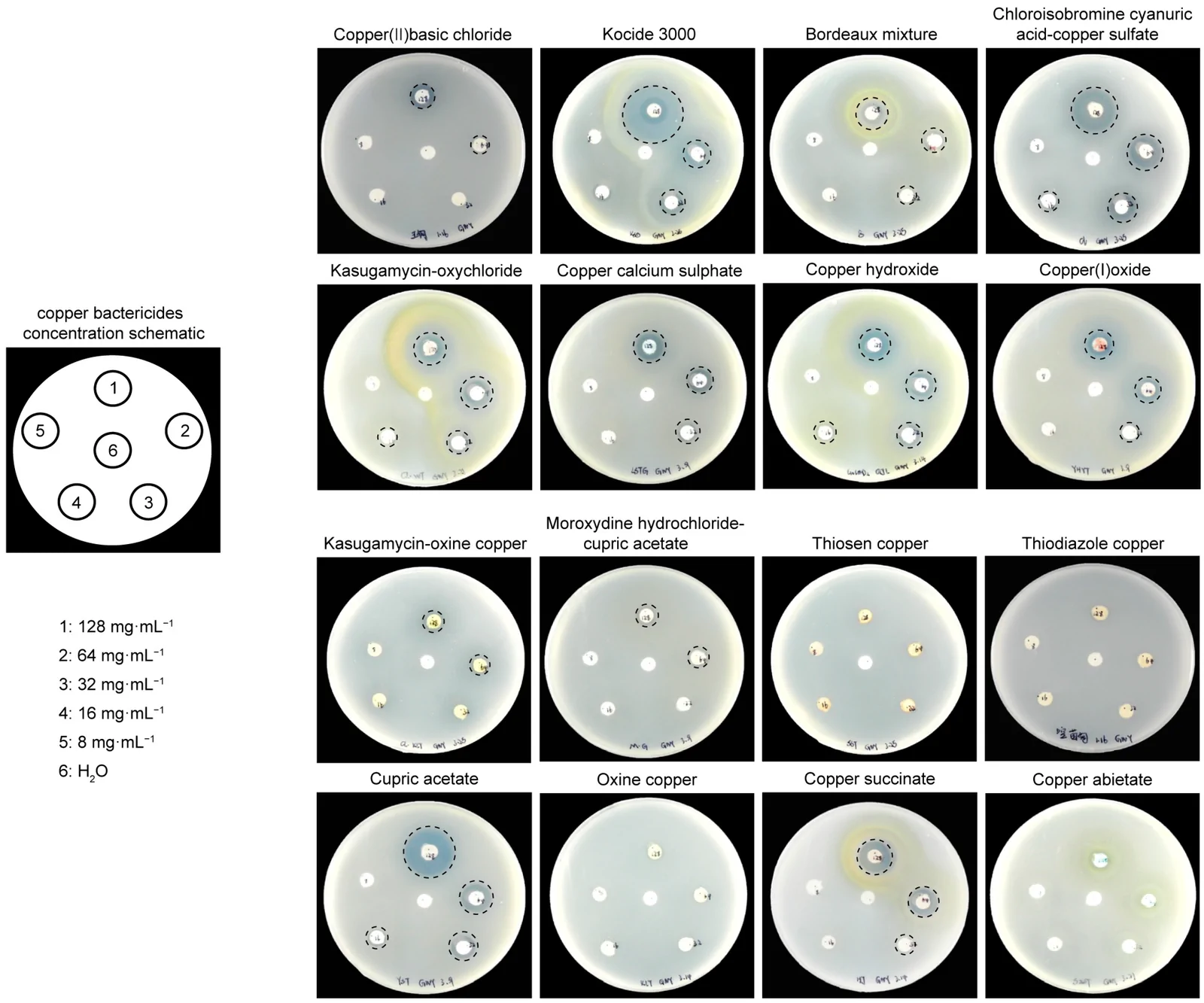
Plate-diffusion screening of commercial copper-containing formulations against *Xanthomonas citri* subsp. *citri* (*Xcc*). Representative inhibition zones around discs containing the tested commercial formulations. Concentrations 1–5 correspond to 128, 64, 32, 16, and 8 mg mL^−1^, respectively; CK, sterile-water control. The black dashed outlines identify the inhibition zones. Values refer to the total mass of each commercial formulation, not to elemental copper concentration.

Sixteen commercial copper-containing formulations were screened against *Xcc* in a plate-diffusion assay (Table 1, Figure 1, and Table S3). At the higher concentrations, relatively large inhibition zones were observed with Kocide 3000, the chloroisobromine cyanuric acid–copper sulfate formulation, cupric acetate, and copper succinate. In contrast, Thiosen Copper, Thiodiazole Copper, oxine copper, and copper abietate produced no visible zone beyond the disc under the same conditions. Because the products differ in copper content, solubility, chemical availability, and co-formulated active ingredients, these screening results compare the commercial formulations as products and cannot be attributed solely to copper species or to an organic/inorganic classification. Four products also contained non-copper active ingredients—chloroisobromine cyanuric acid (d), kasugamycin (e,i), and moroxydine hydrochloride (j)—so their inhibition zones cannot be attributed to copper alone. The plate assay was a single preliminary experiment without independent replication; inhibition-zone values were therefore not statistically compared.

### 2.2. Root-Drench Screening Identifies Formulations That Lower Xcc Populations in Sweet Orange

**Figure 2:**
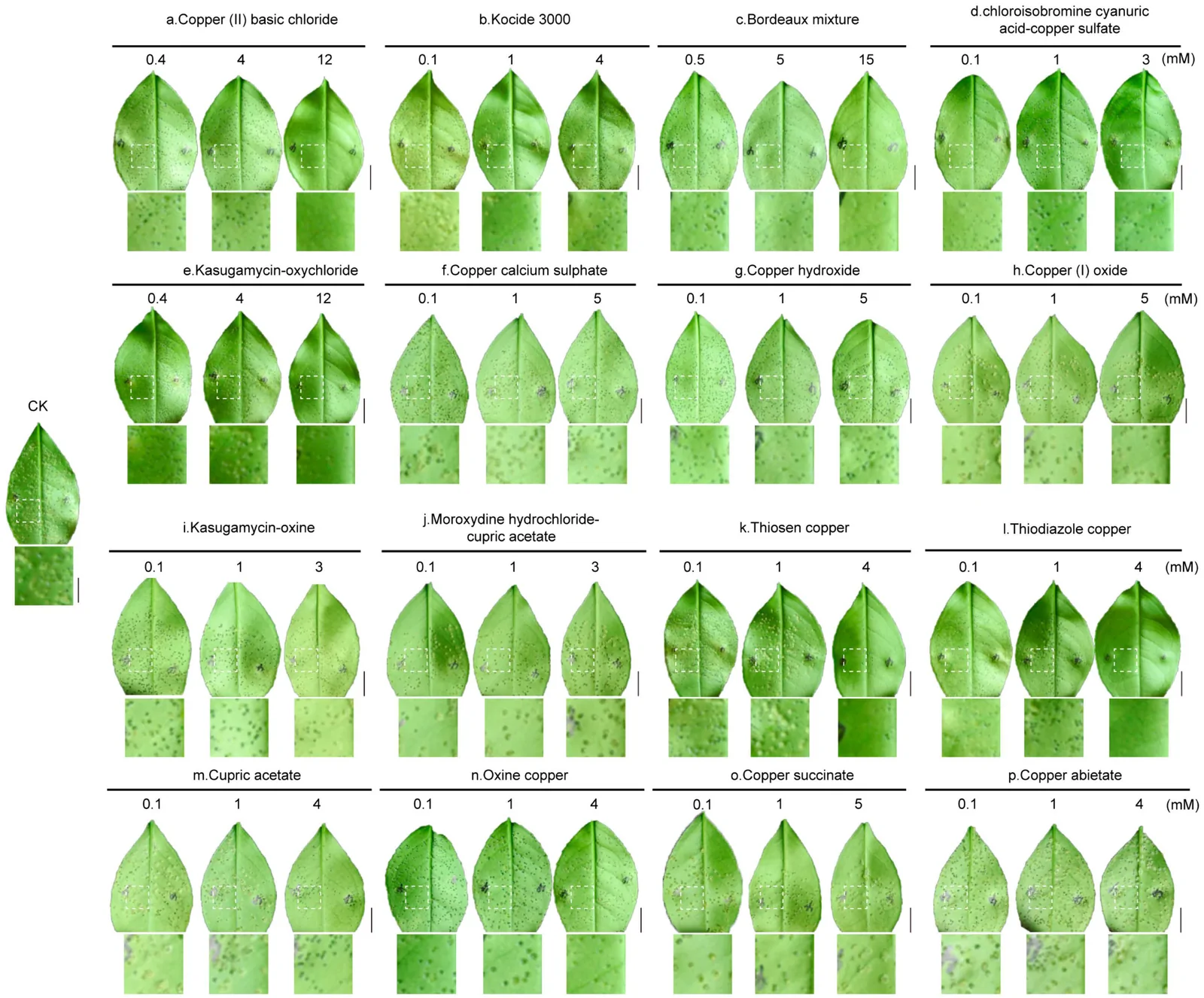
Citrus canker symptoms on the abaxial surface of sweet orange leaves after root treatment with commercial copper-containing formulations. Representative abaxial leaf symptoms at 14 days post-inoculation (dpi) with *Xanthomonas citri* subsp. *citri* (*Xcc*). Formulation codes a–p are defined in Table 1. Numbers above leaves indicate nominal total Cu concentrations (mM), and CK denotes the water control. Dashed rectangles mark regions enlarged below the leaves. Scale bars, 1 cm.

**Figure 3:**
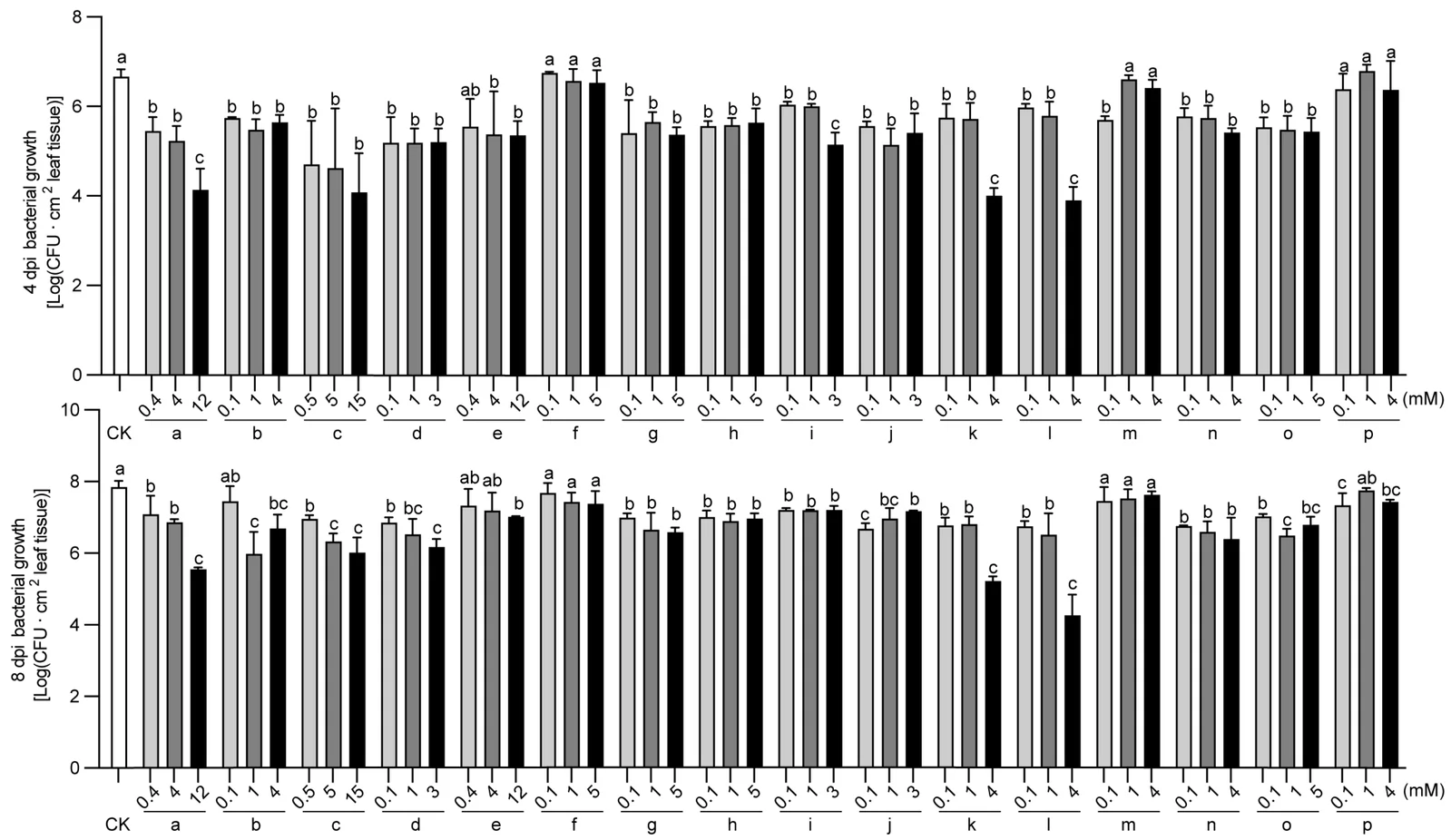
*Xanthomonas citri* subsp. *citri* (*Xcc*) populations in sweet orange leaves after root treatment with commercial copper-containing formulations. *Xcc* populations measured in sweet orange leaves at 4 dpi (upper panel) and 8 dpi (lower panel). Codes a–p refer to Table 1. Treatment levels are expressed as nominal total Cu concentrations (mM). White bars indicate the water control (CK); light-gray, dark-gray, and black bars denote increasing concentration within each formulation. Values are means ± SE (*n* = 3 independently treated pots, each contributing one leaf sample at each time point). Different letters denote significant differences among treatment groups within the same time point (one-way ANOVA followed by Duncan’s new multiple range test, *p* < 0.05). Time points were analyzed separately.

The 16 formulations were next screened by root drenching in sweet orange seedlings at three concentrations per product (Table S4). *Xcc* populations were determined at 4 and 8 dpi, and symptoms were photographed at 14 dpi (Figure 2 and Figure 3 and S1). Several products showed little or inconsistent suppression across the tested concentrations. In contrast, copper(II) basic chloride, Bordeaux Mixture, Thiosen Copper, and Thiodiazole Copper reduced *Xcc* populations at their highest tested concentrations, with Thiosen Copper and Thiodiazole Copper among the lowest at 8 dpi. Lesion-area estimates for the photographs shown are given in Figure S4. We then compared Thiosen Copper and Bordeaux Mixture at 0.3, 0.6, and 1 mM nominal total Cu. Thiosen Copper reduced bacterial growth at the tested concentrations, and its effect was detectable at earlier sampling points than that of Bordeaux Mixture (Figure S2); descriptive lesion-area estimates are shown in Figure S5. Thiosen Copper was chosen for the subsequent analyses because of the contrast between its plate and root-drench results, with Bordeaux Mixture as the conventional comparator.

### 2.3. Thiosen Copper Reduces Xcc Growth in Poncirus Trifoliata

**Figure 4:**
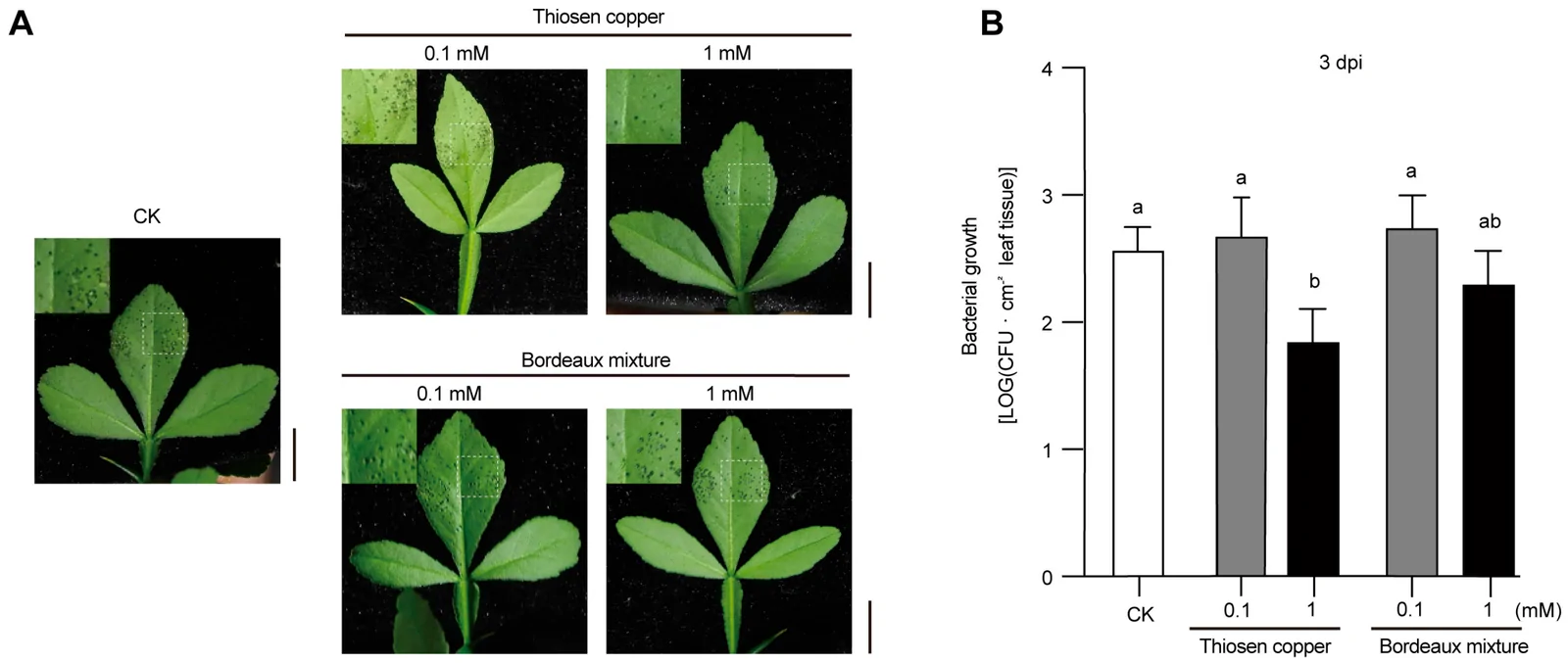
Effects of root drenching with Thiosen Copper and Bordeaux Mixture on citrus canker development in *Poncirus trifoliata*. (**A**) Representative canker symptoms on *Poncirus trifoliata* leaves at 14 days post-inoculation (dpi) after root drenching with Thiosen Copper or Bordeaux Mixture at nominal total Cu concentrations of 0.1 or 1 mM. Insets show enlarged lesion details. Scale bar, 1 cm. (**B**) *Xcc* populations in *P. trifoliata* leaves at 3 dpi. CK denotes the water control. Values are means ± SE (*n* = 3 pots); different letters indicate significant differences among all groups, including CK, at *p* < 0.05 (one-way ANOVA followed by Tukey’s HSD test).

Thiosen Copper and Bordeaux Mixture were also tested in the citrus rootstock *Poncirus trifoliata*. At 1 mM nominal total Cu, Thiosen Copper lowered *Xcc* populations relative to the water control (Figure 4B). At 0.1 mM, Thiosen Copper did not differ from the control. Bordeaux Mixture did not differ from the control at 0.1 or 1 mM, and 1 mM Bordeaux did not differ significantly from 1 mM Thiosen Copper. Representative leaf symptoms are shown in Figure 4A. Lesion-area estimates for the displayed image regions are given in Figure S6.

### 2.4. Differential Effects of Thiosen Copper and Bordeaux Mixture on Chlorophyll Index and Growth Traits in Citrus Seedlings

**Figure 5:**
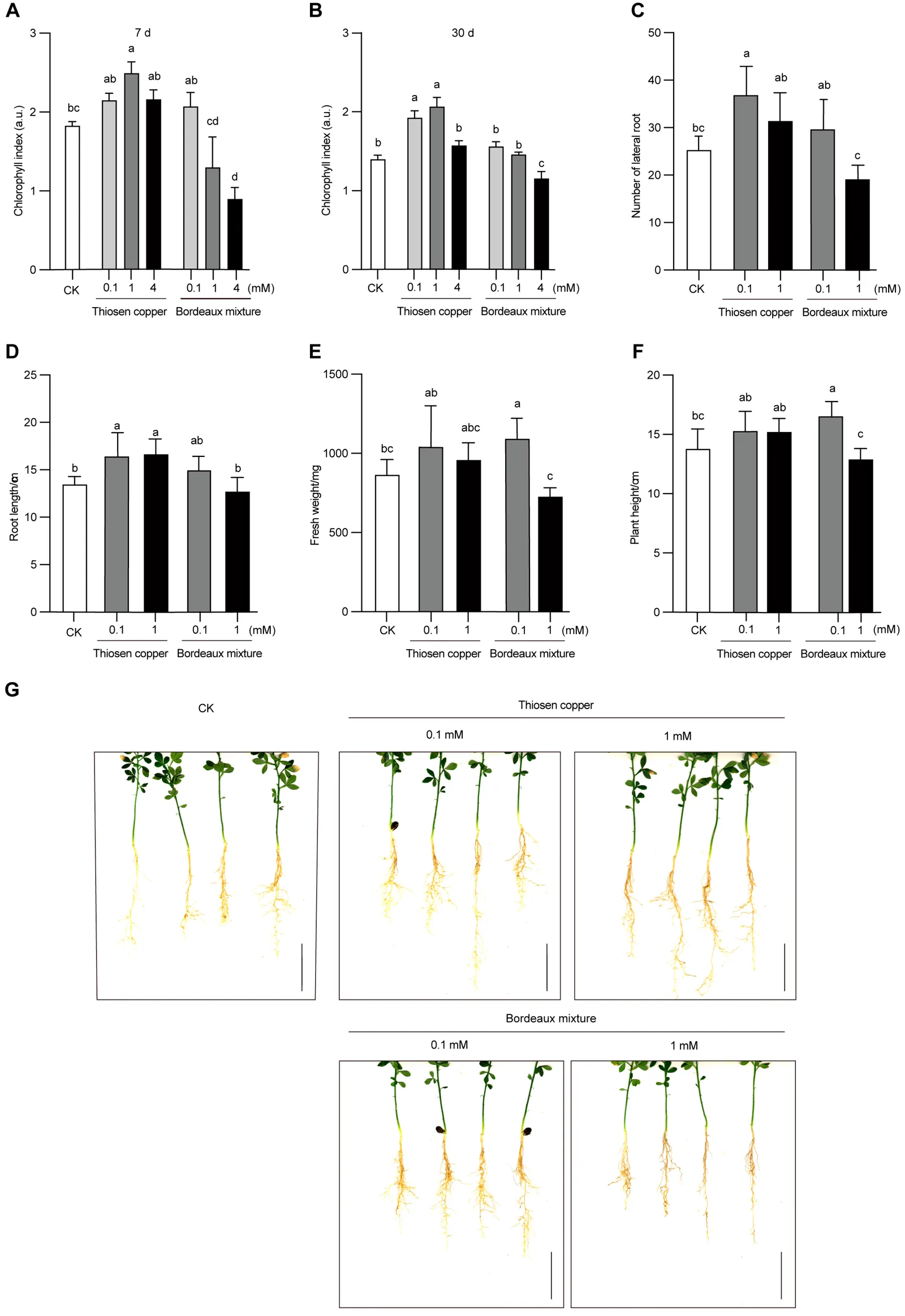
Effects of Thiosen Copper and Bordeaux Mixture on chlorophyll index and growth of citrus seedlings. (**A**,**B**) Chlorophyll index (a.u.) in ‘Dahong’ sweet orange leaves at 7 days (**A**) and 30 days (**B**) after root drenching with Thiosen Copper or Bordeaux Mixture at 0.1, 1, or 4 mM nominal total Cu. Values are reported in arbitrary units without optical path-length correction. Values are means ± SE (*n* = 3 pots). Different letters indicate significant differences among all groups, including CK (one-way ANOVA followed by Tukey’s HSD test, *p* < 0.05). (**C**–**F**) Lateral-root number (**C**), root length (**D**), fresh weight (**E**), and plant height (**F**) in *Poncirus trifoliata* seedlings at 14 days after treatment. Each treatment comprised eight individually potted seedlings (T0.1, *n* = 7 because one value was missing); the water control comprised 12 individually potted seedlings. Values are means ± SE; different letters indicate significant differences among all groups, including CK (one-way ANOVA followed by Tukey’s HSD test, *p* < 0.05). (**G**) Representative *P. trifoliata* root phenotypes at 14 days. Scale bar in G, 5 cm.

We first measured the chlorophyll index in leaves of ‘Dahong’ sweet orange after root treatment. At 7 days, 1 mM Thiosen Copper increased the chlorophyll index relative to the control, whereas 4 mM Bordeaux Mixture reduced it; the other treatments, including 1 mM Bordeaux Mixture, did not differ from the control (Figure 5A). At 30 days, 0.1 and 1 mM Thiosen Copper increased the chlorophyll index relative to the control, whereas 4 mM Bordeaux Mixture reduced it; 4 mM Thiosen Copper and 0.1 and 1 mM Bordeaux Mixture did not differ from the control (Figure 5B).

Growth traits were examined separately in *Poncirus trifoliata* at 14 days after treatment. At 0.1 mM, Thiosen Copper increased lateral-root number and root length, and Bordeaux Mixture increased fresh weight and plant height, relative to the control; the other traits did not differ from the control (Figure 5C–F). At 1 mM, Thiosen Copper also increased root length relative to the control. Bordeaux Mixture at 1 mM reduced lateral-root number, primary-root length, fresh weight, and plant height relative to 0.1 mM Thiosen Copper. It also reduced lateral-root number, fresh weight, and plant height relative to 0.1 mM Bordeaux Mixture, and lateral-root number, root length, and plant height relative to 1 mM Thiosen Copper. These Bordeaux Mixture reductions did not differ significantly from the control (Figure 5C–F).

### 2.5. Tissue Copper Accumulation Following Thiosen Copper and Bordeaux Mixture Treatments

**Figure 6:**
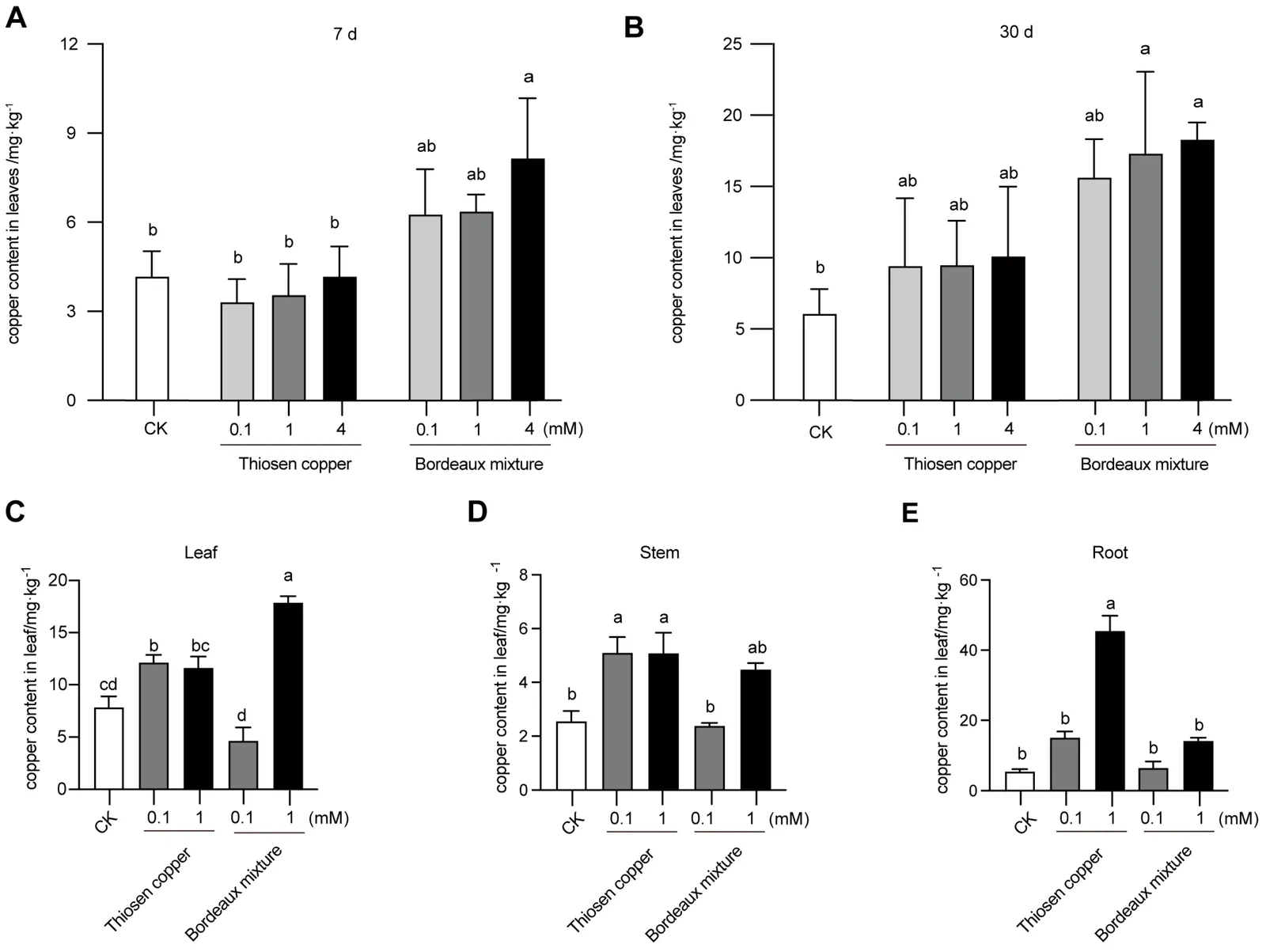
Total copper accumulation in citrus tissues following root drenching. (**A**,**B**) Total Cu in ‘Dahong’ sweet orange leaves at 7 days (**A**) and 30 days (**B**) after root drenching with Thiosen Copper or Bordeaux Mixture at nominal total Cu concentrations of 0.1, 1, or 4 mM. Values are means ± SE (*n* = 3 pots). Different letters indicate significant differences among all groups, including CK (one-way ANOVA followed by Tukey’s HSD test, *p* < 0.05). (**C**–**E**) Total Cu in leaves (**C**), stems (**D**), and roots (**E**) of *Poncirus trifoliata* seedlings at 7 days after treatment with 0.1 or 1 mM nominal total Cu. CK denotes the water control and is included once: *n* = 4 for leaf, 4 for stem, and 3 for root; treatment *n* = 3–4 (exact *n* in Table S5). Values are means ± SE; different letters indicate significant differences among groups (one-way ANOVA followed by Tukey’s HSD, *p* < 0.05).

Total Cu was measured in leaves of ‘Dahong’ sweet orange at 7 and 30 days after root treatment. At 7 days, 4 mM Bordeaux Mixture increased leaf Cu relative to the control and to 4 mM Thiosen Copper; 0.1 and 1 mM Bordeaux Mixture did not differ from the control, and none of the Thiosen Copper concentrations increased leaf Cu (Figure 6A). At 30 days, 1 and 4 mM Bordeaux Mixture increased leaf Cu relative to the control; 0.1 mM Bordeaux Mixture and the Thiosen Copper treatments did not differ from the control, and 4 mM Bordeaux Mixture did not differ significantly from 4 mM Thiosen Copper (Figure 6B).

In a separate experiment, total Cu was measured in leaves, stems, and roots of *Poncirus trifoliata* at 7 days after root drenching. In leaves, 1 mM Bordeaux Mixture had the highest Cu and exceeded all other groups. The 0.1 mM Thiosen Copper was higher than CK and 0.1 mM Bordeaux Mixture; 1 mM Thiosen Copper was higher than 0.1 mM Bordeaux Mixture but did not differ from CK or 0.1 mM Thiosen Copper; CK and 0.1 mM Bordeaux Mixture did not differ (one-way ANOVA, *p* < 0.001; Figure 6C). In stems, 0.1 and 1 mM Thiosen Copper increased Cu relative to CK and 0.1 mM Bordeaux Mixture; 1 mM Bordeaux Mixture did not differ from any group (*p* = 0.00165; Figure 6D). In roots, 1 mM Thiosen Copper increased Cu relative to all other groups; no other pairwise difference was significant (*p* < 0.001; Figure 6E).

### 2.6. Copper-Responsive Expression of PtCOPT Genes and Candidate Role of Citrus COPT Genes in Response to Xcc Infection

**Figure 7:**
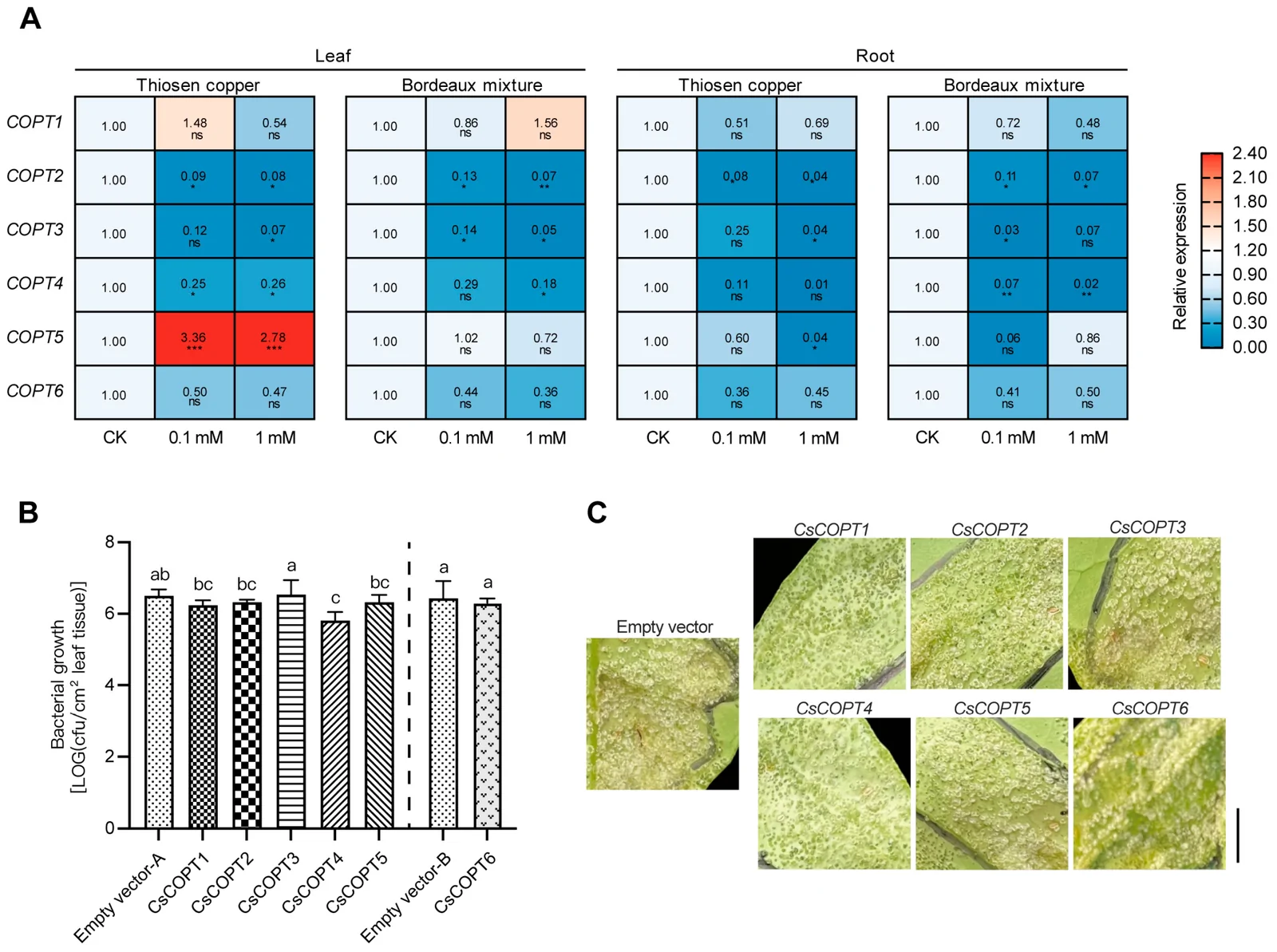
Copper-responsive expression of *PtCOPT* genes in *Poncirus trifoliata* and candidate role of *CsCOPT* genes in sweet orange during *Xcc* infection. (**A**) Heatmap of *PtCOPT1*–*PtCOPT6* transcript levels in leaves and roots of *Poncirus trifoliata* at 7 days after root treatment with Thiosen Copper or Bordeaux Mixture (0.1 or 1 mM nominal total Cu). Each formulation had its own water control; values are mean normalized expression relative to the matched water control (CK = 1); the color scale is 0–2.40, with three biological replicates per gene × tissue × treatment. Statistical comparisons were performed on log2(normalized expression). Stars indicate significant differences from the matched CK by Dunnett’s test, with Benjamini–Hochberg correction within tissue and formulation (* *p* < 0.05, ** *p* < 0.01, *** *p* < 0.001); stars are shown only when the gene-level one-way ANOVA was significant after BH correction. Exact means, SE, *n*, and *p* values are shown in Table S6. (**B**) *Xcc* populations in sweet orange leaves at 5 dpi after transient overexpression of *CsCOPT1*–*CsCOPT6* or the empty-vector control (OE-EV). Values are means ± SE (*n* = 3 plants; one value per plant per construct). Leaf A: EV-A and *CsCOPT1*–*CsCOPT5*; Leaf B: EV-B and *CsCOPT6*. EV-A and EV-B were ground separately and used as the matched controls for their respective leaves. Letters are comparable only within each leaf section. For Leaf A, different letters indicate significant differences among EV-A and *CsCOPT1*–*CsCOPT5* (randomized complete block ANOVA with plant as a block, followed by Tukey’s HSD, *p* < 0.05); for Leaf B, EV-B and *CsCOPT6* share the same letter (paired *t*-test, *p* = 0.718). (**C**) Representative leaf symptoms at 14 dpi; symptoms were not quantitatively scored. Scale bar, 1 cm.

We measured *PtCOPT* transcript levels in leaves and roots of *Poncirus trifoliata* 7 days after root treatment with Thiosen Copper or Bordeaux Mixture. Each formulation had its own water control, and the two controls were not pooled. Figure 7A displays mean normalized expression; statistical tests were performed on log2-transformed values. Normalized expression was analyzed separately for each gene, tissue, and formulation by one-way ANOVA across CK, 0.1, and 1 mM (*n* = 3 biological replicates), followed by Dunnett’s test against the matched CK with Benjamini–Hochberg correction. In leaves, under Thiosen Copper, *COPT2* and *COPT4* were lower at 0.1 and 1 mM, *COPT3* was lower at 1 mM, and *COPT5* was higher at 0.1 and 1 mM; under Bordeaux Mixture, *COPT2* and *COPT3* were lower at 0.1 and 1 mM, and *COPT4* was lower at 1 mM. In roots, under Thiosen Copper, *COPT2* was lower at 0.1 and 1 mM, and *COPT3* and *COPT5* were lower at 1 mM; under Bordeaux Mixture, *COPT2* was lower at 0.1 and 1 mM, *COPT3* was lower at 0.1 mM, and *COPT4* was lower at 0.1 and 1 mM. *COPT1* and *COPT6* showed no significant response in either tissue. Exact means, SE, *n*, and *p* values are shown in Table S6.

To assess whether the corresponding sweet orange *COPT* genes affect *Xcc* growth, we transiently overexpressed *CsCOPT1*–*CsCOPT6* individually in sweet orange leaves. Each construct increased the transcript level of its target gene relative to the empty-vector control (Figure S3). Among the six genes, only *CsCOPT4* overexpression was associated with significantly lower bacterial populations (Figure 7B). In the matched-control analysis, the Leaf A treatment effect was significant (F(5,10) = 9.36, *p* = 0.0016); *CsCOPT4* was lower than EV-A (Dunnett *p* = 0.0075), whereas *CsCOPT3* did not differ from EV-A (*p* = 0.138). *CsCOPT6* did not differ from EV-B (paired *t*-test, *p* = 0.718). Figure 7C is representative and was not quantitatively scored.

## 3. Discussion

### 3.1. Thiosen Copper Suppresses Xcc in Planta Despite Showing No Inhibition Zone in Plate Assays

The mismatch between the plate and root-drench assays was clearest for Thiosen Copper, which produced no visible inhibition zone around the filter-paper discs yet lowered *Xcc* populations after root application. Formulation properties affecting dispersion and diffusion through agar may help explain this difference. The root-drench response may also involve changes in the host. In kiwifruit, for example, enhanced lignification has been linked to tolerance to bacterial canker [25]. The 16-product screen should be interpreted as a comparison of commercial formulations under the tested conditions, not as a controlled comparison of copper chemical forms: the products differed in nominal Cu content, solubility, chemical availability, and co-formulated active ingredients. Four products contained non-copper active ingredients (chloroisobromine cyanuric acid, kasugamycin, or moroxydine hydrochloride), so their effects cannot be attributed to copper alone. In addition, the tested Cu concentrations were not identical across products (Table S4), so efficacy at the highest tested concentration should not be interpreted as an intrinsic product ranking. Our study tested *Xcc* strain DL509. The infiltration assay bypassed natural bacterial entry, and orchard performance remains to be evaluated under varying weather and exposure [10].

### 3.2. Physiological and Tissue Cu Responses to Thiosen Copper and Bordeaux Mixture

The two formulations produced different physiological responses, which also depended on host species and sampling time. In sweet orange, Thiosen Copper increased the leaf chlorophyll index in several treatments without a significant increase in bulk leaf Cu, whereas Bordeaux Mixture led to greater leaf Cu accumulation and, at higher concentrations, lower chlorophyll index. Chlorophyll index is one physiological indicator; photosynthetic responses also depend on environmental conditions such as heat [26]. Excess copper can disrupt plant metabolism and growth [27,28,29,30]. Total tissue Cu measurements may include surface-retained Cu and do not resolve uptake or redistribution rates. Environmental effects require assessment under practical application conditions.

### 3.3. CsCOPT4 Overexpression Is Associated with Reduced Xcc Growth

Studies in *Arabidopsis* link metal transport to plant defense: *YSL3* participates in responses to *Pseudomonas syringae* [31], and *HMA2* and *HMA4* mediate zinc accumulation associated with resistance to a necrotrophic fungus [32]. In this study, root-applied copper formulations altered *PtCOPT* expression patterns in *Poncirus trifoliata*, whereas the corresponding sweet orange *COPT* genes were assessed separately by transient overexpression. These experiments involve different species and experimental systems; the gene pairs are matched by their identifiers and annotations (Table S2). In an unrooted maximum-likelihood tree of the 12 citrus *COPT* proteins, five pairs (*COPT2*–*COPT6*) formed supported sister pairs (Figure S7); *COPT1* did not form a supported pair, and its correspondence remains annotation-based. *PtCOPT5* showed a treatment-associated expression pattern, yet *CsCOPT5* overexpression produced no clear resistance phenotype in sweet orange. Because amplification efficiency was not experimentally determined, small differences in *PtCOPT* transcript abundance should be interpreted cautiously. Only *CsCOPT4* overexpression was associated with lower *Xcc* populations. These results identify *CsCOPT4* as a candidate for further study of citrus defense. Its contribution to Thiosen Copper-mediated protection remains to be established.

## 4. Materials and Methods

### 4.1. Plant and Bacterial Materials

Seedlings of *Poncirus trifoliata* and ‘Dahong’ sweet orange were grown in a greenhouse at 28 ± 2 °C and ~80% relative humidity under a 16:8 h light:dark photoperiod. The *Xcc* strain used was *DL509*, originally isolated as described previously [33]. Both species were grown in perlite in pots measuring 10 × 10 × 19 cm (length × width × height). Sweet orange seedlings were four months old and approximately 40 cm tall at treatment; P. trifoliata seedlings were two to three months old. One week before treatment, plants were watered thoroughly, and pots were checked to have comparable moisture at treatment.

### 4.2. In Vitro Plate Assay Against Xcc

LB agar was melted and cooled to 45 °C before 6 mL of an *Xcc* suspension (10^9^ CFU mL^−1^) was mixed with 60 mL of the medium. The inoculated medium was poured into Petri dishes and allowed to solidify. Aqueous preparations of each commercial formulation were made at 128, 64, 32, 16, and 8 mg mL^−1^, expressed as total formulation mass; sterile water was used as the control. For each concentration, 5 μL was applied to a sterile 6-mm filter-paper disc placed on the inoculated medium. After 48 h at 30 °C, inhibition-zone diameters were measured by the cross-measurement method. The assay was used to screen commercial products because inhibition-zone size reflects both antimicrobial activity and formulation-dependent solubility and diffusion. The assay was a single preliminary experiment: each product × concentration was tested on one plate with one filter-paper disc, and no independent replication was performed. Inhibition-zone diameters are therefore single measurements and were not statistically compared.

### 4.3. Root-Drench Treatment and Xcc Growth Assay

Commercial formulations were applied by root drenching to potted ‘Dahong’ sweet orange seedlings. For both sweet orange and *Poncirus trifoliata*, 100 mL of treatment solution was applied per seedling. Each treatment comprised three independently treated pots with four seedlings per pot, corresponding to 400 mL per pot. Treatment concentrations are reported as nominal total Cu concentrations (mM), calculated from the total elemental Cu content of the copper-bearing ingredients; they do not represent free Cu^2+^ concentrations. For a mass-based formulation, *C*(Cu) (mM) = 1000 × *m*(formulation) (g) × Σ[*a*(*j*) × *f*(Cu,*j*)]/[63.546 × *V*(solution) (L)], where *a*(*j*) is the mass fraction of copper-bearing ingredient *j*, *f*(Cu,*j*) is its elemental Cu mass fraction, and *V* is the final solution volume. For ingredients declared in g L^−1^, ingredient mass was calculated from the declared concentration and formulation volume. Non-copper active ingredients were excluded from the calculation. For Bordeaux Mixture, the calculation used an 80% (*w*/*w*) copper sulfate content on a CuSO_4_·5H_2_O basis (molar mass, 249.68 g mol^−1^), giving an elemental Cu mass fraction of 0.80 × 63.546/249.68 = 0.2036 (20.36%, *w*/*w*). Each formulation was screened at three concentrations. Screening concentrations were converted from label-recommended field rates to nominal total Cu and were not limited by formulation solubility. From the plate and root-drench screens, 1 mM was selected as the anchor because a preliminary trial showed strong efficacy at 1 mM; 0.1 and 4 mM were used as lower and higher boundaries in sweet orange, and 0.1 and 1 mM were used for *P. trifoliata* because 1 mM Bordeaux Mixture already reduced growth. The highest concentration was a high-dose boundary, not an optimum. The screening concentrations and calculated nominal total Cu inputs per seedling and per pot are listed in Table S4. These concentrations were used as the tested screening levels; because the nominal total Cu levels differed among formulations, comparisons at each product’s highest tested concentration do not establish an intrinsic ranking of formulation efficacy. One week before treatment, plants were watered thoroughly, and pots were checked to have comparable moisture at treatment. Fourteen days after root drenching, leaves were infiltrated with an *Xcc* suspension (10^4^ CFU mL^−1^). At 4 and 8 days post-inoculation (dpi), 6-mm discs were excised from inoculated leaves, homogenized in sterile water, and serially diluted, and 5 μL aliquots were spotted onto LB medium. After incubation at 30 °C for 48 h, colony counts were recorded. Lesion development was photographed at 14 dpi. In the focused sweet orange comparison of Thiosen Copper and Bordeaux Mixture at 0.3, 0.6, and 1 mM nominal total Cu, bacterial populations were sampled at 3, 5, and 10 dpi (Figure S2B). In the *P. trifoliata* comparison at 0.1 and 1 mM nominal total Cu, bacterial populations were sampled at 3 dpi (Figure 4B). The same inoculation concentration was used for sweet orange and *P. trifoliata*.

### 4.4. Determination of Plant Growth Parameters

Growth traits were assessed in *Poncirus trifoliata* seedlings 14 days after root drenching with Thiosen Copper or Bordeaux Mixture at nominal total Cu concentrations of 0.1 and 1 mM. The seedlings were raised from seed, were two to three months old, and were grown in the same potting system as the sweet orange plants. Each treatment comprised eight individually potted seedlings (T0.1, *n* = 7 because one value was missing), and the water control comprised 12 individually potted seedlings; each pot was treated as one experimental unit, and no averaging across seedlings was performed. At 14 days, roots were rinsed with water and gently blotted to remove surface moisture. All visibly distinguishable lateral roots were counted, with no minimum length threshold. Primary-root length was measured from the root collar to the tip, fresh weight was recorded immediately after blotting, and plant height was measured from the root collar to the shoot apex.

### 4.5. Determination of Leaf Chlorophyll Index

Chlorophyll was extracted with ethanol. Fully expanded leaves of ‘*Dahong*’ sweet orange were sampled at 7 and 30 days after root drenching with Thiosen Copper or Bordeaux Mixture. For each sample, 0.1 g of chopped leaf tissue was extracted with 10 mL of 96% ethanol in darkness for 24 h, with gentle inversion 4–5 times during extraction. A 200 μL aliquot of extract was transferred to a transparent flat-bottom 96-well polystyrene plate (Bioland, Hangzhou, China; Cat. No. CCP06N-096PS), and absorbance was measured at 665 and 649 nm on a Tecan Infinite M200 PRO microplate reader (Tecan Austria GmbH, Grödig, Austria), using the same instrument settings for all samples. No separate path-length calibration was applied. Because the same plate type, volume, and instrument settings were used throughout, the nominal optical path length was constant across treatments; the index is intended for comparisons among treatments within each sampling time under identical measurement conditions, but values are not absolute 1-cm concentrations. A chlorophyll index was calculated from the measured absorbances using the coefficients of Lichtenthaler and Wellburn [34], derived for 96% (*v*/*v*) ethanol, and the fixed extraction ratio (10 mL per 0.1 g), index = (0.663A665 + 1.808A649) × D, where D is the dilution factor and A665 and A649 are the measured absorbances. The index is reported in arbitrary units (a.u.) and is not an absolute chlorophyll concentration. Chlorophyll index is reported in Figure 5A,B.

### 4.6. Determination of Plant Copper Content

Leaves of ‘Dahong’ sweet orange were collected at 7 and 30 days after root drenching. In a separate experiment, leaves, stems, and roots of *Poncirus trifoliata* were sampled at 7 days (Figure 6). The tissues were rinsed with distilled water, heated at 105 °C for 30 min in a forced-air oven, and then dried at 65 °C to constant weight. Dried tissue from each organ was ground to a powder, and 0.5 g was digested on a hot plate with 10.0 mL of HNO_3_–HClO_4_ (4:1, *v*/*v*). The digest was heated at 200 °C until dense white fumes appeared and the digest was clear. After cooling, the digest was transferred with 2% HNO_3_ and brought to a final volume of 50 mL. Total Cu was measured by inductively coupled plasma atomic emission spectroscopy (ICP-AES; Agilent 5110, Agilent Technologies, Santa Clara, CA, USA) against an external calibration series of 0, 0.2, 0.8, 1.0, 1.5, and 2.0 μg mL^−1^ Cu, with a reagent blank. Tissue Cu concentrations are expressed on a dry-weight basis.

### 4.7. Quantitative Gene Expression Analysis

Total RNA was extracted with TRIzol Reagent (Invitrogen, Carlsbad, CA, USA; Cat. No. 15596026) from leaves and roots of Poncirus trifoliata seedlings at 7 days after root treatment with Bordeaux Mixture, Thiosen Copper, or sterile water. RNA integrity was checked by agarose-gel electrophoresis, and first-strand cDNA was synthesized using M-MLV RT Premix (Accurate Biology, Changsha, China; Cat. No. AG11706). Quantitative RT-PCR was performed with SYBR Green Master Mix (Vazyme, Nanjing, China; Cat. No. Q711-02) on a Bio-Rad CFX96 instrument (Bio-Rad Laboratories, Hercules, CA, USA). Three biological replicates were analyzed, each with three technical replicates. EF1α was used as the reference gene, and relative transcript levels were calculated by the 2^−ΔΔCt^ method [35]. Primer sequences are provided in Table S1. The COPT genes assayed in P. trifoliata are designated PtCOPT1–PtCOPT6, and the corresponding sweet orange genes are CsCOPT1–CsCOPT6; gene identifiers for both species are listed in Table S2. Primer specificity was verified by BLAST (v2.14.0); amplification efficiency was not experimentally determined. No efficiency correction was applied, and the 2−ΔΔCt values assume comparable amplification efficiency. EF1α Cq stability was assessed across the 36 samples from the original qPCR run; Cq did not differ significantly among treatments (one-way ANOVA, *p* = 0.917 for leaves and *p* = 0.984 for roots), and technical replicates differed by 0.20–0.22 Cq on average. Within-group CV was 6.9% for leaves and 10.3% for roots.

### 4.8. Transient Overexpression in Citrus Leaves

The six sweet orange genes *CsCOPT1*–*CsCOPT6* were individually cloned into pCAMBIA1300 and introduced into *Agrobacterium tumefaciens* strain EHA105. Suspensions carrying each overexpression construct or the empty vector (OD_600_ = 0.6) were infiltrated into ‘Dahong’ sweet orange leaves. After 24 h, the leaves were infiltrated with an *Xcc* suspension (10^5^ CFU mL^−1^). Target-gene transcript levels were measured by qRT-PCR in samples collected at 2 dpi to verify transient expression. *Xcc* populations were determined at 5 dpi, and disease symptoms were photographed at 14 dpi. For Figure 7B, three independent plants were used. On each plant, Leaf A was co-infiltrated with *CsCOPT1*–*CsCOPT5* plus EV-A, and Leaf B was infiltrated with *CsCOPT6* plus EV-B. EV-A and EV-B were ground separately and used as the matched controls for their respective leaves: EV-A for *CsCOPT1*–*CsCOPT5* and EV-B for *CsCOPT6*. Each construct was therefore tested in three plants (*n* = 3 biological replicates). Injection sites within a leaf were technical replicates and were averaged before analysis. Figure 7C is representative. For Figure S3, independent groups of three plants were infiltrated with each construct or the matched empty vector; these samples were used for the 2-dpi qRT-PCR validation and were not paired by plant.

### 4.9. Statistical Analysis

Statistical analyses were performed in R 4.4.2. Data are presented as means ± SE. For Figure 4B, Figure 5A–F and Figure 6A–E, the water control was included once as a treatment group, and groups were compared by one-way ANOVA followed by Tukey’s HSD test (Tukey–Kramer for unequal *n*). Normality and homogeneity of variance were checked with Shapiro–Wilk and Levene’s tests; the tests did not detect significant deviations from normality or variance homogeneity in any of the 12 panels. For Figure 7A, each formulation had its own water control; normalized expression was log2-transformed and analyzed separately for each gene, tissue, and formulation by one-way ANOVA across CK, 0.1, and 1 mM, followed by Dunnett’s test against the matched CK. ANOVA *p* values were Benjamini–Hochberg-adjusted across the six *COPT* genes within each tissue and formulation, and Dunnett *p* values were adjusted across gene × treatment within each tissue and formulation. Figure 7B was analyzed with plant as a block. For Leaf A, EV-A and *CsCOPT1*–*CsCOPT5* were compared by randomized complete block ANOVA with plant as a block, followed by Tukey’s HSD; Dunnett’s test was used for comparisons with EV-A. For Leaf B, *CsCOPT6* was compared with EV-B by a paired *t*-test across the three plants. Each construct had one value per plant; Figure 3 was analyzed by one-way ANOVA followed by Duncan’s test at each time point. Figure S2B was analyzed separately at each sampling time. For Figure S3, independent groups of three plants were used for each construct or EV; each construct was compared with its matched EV using a two-tailed Welch’s unpaired *t*-test on ΔCt. Each panel represents a single pre-specified construct–EV comparison, and no correction for multiple comparisons across genes was applied. Differences were considered significant at *p* < 0.05.

For root-drench experiments, the independently treated pot was the experimental unit. Bacterial, chlorophyll, and leaf Cu measurements used three pots per treatment with four seedlings per pot; measurements from seedlings in the same pot were averaged. Growth traits used eight individually potted seedlings per treatment (T0.1, *n* = 7 because one value was missing) and 12 water-control pots; each pot was one experimental unit, and no averaging across seedlings was performed. For bacterial-population assays, one leaf was collected per pot at each time point (*n* = 3 pots per treatment); two discs per leaf were pooled, and repeated plating served as technical replication. In the commercial-formulation screen, the same plants were sampled at 4 and 8 dpi; each time point was analyzed separately, with each formulation-by-concentration combination treated as a separate group. In the focused experiment shown in Figure S2B, the same plants were sampled at 3, 5, and 10 dpi; each time point was analyzed separately. For Figure 7B, *n* = 3 refers to three plants; each plant contributed both leaves (Leaf A: five constructs plus EV-A; Leaf B: *CsCOPT6* plus EV-B), and EV-A and EV-B were ground separately and used as the matched controls for their respective leaves. Each construct had one value per plant. For Figure S3, *n* = 3 refers to three independent plants per construct or EV group; EV and construct samples were not paired, and each construct was compared with its matched EV by a two-tailed Welch’s unpaired *t*-test on ΔCt. Injection sites within a leaf were technical replicates.

### 4.10. Image-Based Estimation of Lesion Area

Lesion area (%) was estimated with ImageJ (version 1.54g) in the magnified leaf regions shown in Figure 2, Figure S2A and Figure 4A. Dark pixels were segmented within each region, and lesion area was calculated as the dark-pixel area divided by the leaf-tissue area of that region, multiplied by 100. The primary relative dark-contrast threshold was 24/255, and thresholds of 20/255 and 28/255 were tested for sensitivity. In total, 49 regions from Figure 2, seven from Figure S2A, and five from Figure 4A were analyzed, with one displayed region per treatment. These image-derived estimates describe the displayed regions and are not independent biological replicates.

### 4.11. Phylogenetic Analysis

Protein sequences of *PtCOPT1*–*PtCOPT6* and *CsCOPT1*–*CsCOPT6* (12 sequences) were aligned, and the 667-amino-acid alignment was analyzed in IQ-TREE 3.0.1. ModelFinder selected the VT + G4 model according to the Bayesian information criterion. A maximum-likelihood tree was inferred with 1000 ultrafast bootstrap replicates (random seed 991264), and the tree was displayed as an unrooted radial tree. No outgroup was included. Bootstrap support was mapped to node symbols: black, ≥90%; grey, 70–89%.

## 5. Conclusions

Of the 16 commercial formulations examined, Thiosen Copper produced no visible inhibition zone in the plate-diffusion assay but reduced *Xcc* populations after root application. Thiosen Copper and Bordeaux Mixture also differed in their effects on chlorophyll index, seedling growth, and tissue Cu concentrations in the citrus species tested. Transient overexpression of *CsCOPT4* in sweet orange leaves was accompanied by reduced *Xcc* growth, identifying this gene as a candidate for further study.

## Figures and Tables

**Table 1:** Commercial copper-containing formulations used in the plate-diffusion screening assay.

Code	Commercial Formulation	Declared Ingredients/Elemental Cu	Type	Manufacturer
a	Copper(II) basic chloride	Copper(II) basic chloride 30%; Cu ~17.9%	SC	Qingdao Dongsheng Pharmaceutical Co., Ltd., Qingdao, China
b	Kocide 3000	Copper hydroxide 46.1%; Cu 30.0% (label)	WG	Certis USA LLC (U.S. label), Columbia, MD, USA
c	Bordeaux Mixture	Copper sulfate 80% (CuSO_4_·5H_2_O basis); Cu ~20.36%	WP	Cerexagri (USA), Puteaux, France
d	Chloroisobromine cyanuric acid + copper sulfate	Chloroisobromine cyanuric acid 50%; copper sulfate 2%; Cu 0.51–0.80%	SP	Nanjing Nannong Pesticide Technology Development Co., Ltd., Nanjing, China
e	Kasugamycin + copper oxychloride	Copper oxychloride 29.5%; kasugamycin 0.5%; Cu ~17.6%	WP	Jiangxi Zhonghe Chemical Co., Ltd., Jiujiang, China
f	Copper calcium sulfate	Copper calcium sulfate 660 g/L; Cu ~170 g/L	SC	Sichuan Zhanpeng Fengying Biotechnology Co., Ltd., Meishan, China
g	Copper hydroxide	Copper hydroxide 46%; Cu ~30.0%	SC	DuPont (USA), Wilmington, DE, USA
h	Copper(I) oxide	Copper(I) oxide 86.2%; Cu ~76.6%	WP	NORDOX AS, Oslo, Norway
i	Kasugamycin + oxine copper	Oxine copper 33%; kasugamycin 3%; Cu ~6.0%	SC	Anhui Bohai Biotechnology Co., Ltd., Lu’an, China
j	Moroxydine hydrochloride + cupric acetate	Moroxydine hydrochloride 16%; cupric acetate 4%; Cu ~1.4%	WP	Jiangsu Kesheng Group Co., Ltd., Yancheng, China
k	Thiosen Copper	Thiosen copper 30%; Cu ~5.6%	SC	Zhejiang Dongfeng Chemical Co., Ltd., Wenzhou, China
l	Thiodiazole Copper	Thiodiazole copper 20%; Cu ~3.9%	SC	Zhejiang Longwan Chemicals Co., Ltd., Wenzhou, China
m	Cupric acetate	Cupric acetate 20%; Cu ~7.0%	WP	Weifang Shuangxing Pesticide Co., Ltd., Weifang, China
n	Oxine copper	Oxine copper 33.5%; Cu ~6.1%	SC	Shanghai Hulian Biological Pharmaceutical (Xiayi) Co., Ltd., Shangqiu, China
o	Copper succinate	Copper succinate 30%; Cu ~10.6%	WP	Hebei Zhongbao Green Crop Technology Co., Ltd., Langfang, China
p	Copper abietate	Copper abietate 20%; Cu ~1.9%	SC	Qingdao Haina Biotechnology Co., Ltd., Qingdao, China

SC, suspension concentrate; WP, wettable powder; WG, water-dispersible granules; SP, soluble powder. Declared ingredient contents are taken from product labels. Cu values preceded by ~ are stoichiometric estimates; the Kocide 3000 value is label-reported. The Bordeaux Mixture estimate uses 80% copper sulfate on a CuSO_4_·5H_2_O basis. The Cu range for the chloroisobromine cyanuric acid–copper sulfate formulation reflects an unspecified copper sulfate hydration state. The estimate for copper calcium sulfate assumes CuSO_4_·3Cu(OH)_2_·3CaSO_4_·7H_2_O.

## Data Availability

The data supporting the findings of this study are available within the article and its Supplementary Materials. Additional data are available from the corresponding authors upon reasonable request.

## Supplementary Materials

The following supporting information can be downloaded at https://www.mdpi.com/article/10.3390/plants15213453/s1, Figure S1: Additional photographs of citrus canker symptoms on sweet orange leaves after root drenching with the tested commercial formulations; Figure S2: Lesion-area images and time-course Xcc populations in sweet orange leaves after root drenching with Thiosen Copper and Bordeaux Mixture; Figure S3: Transcript levels of CsCOPT genes after transient overexpression in sweet orange leaves; Figure S4: Lesion-area estimates for the leaf regions shown in Figure 2; Figure S5: Descriptive lesion-area estimates for the Thiosen Copper–Bordeaux Mixture comparison; Figure S6: Lesion-area estimates for the leaf regions shown in Figure 4; Figure S7: Unrooted maximum-likelihood tree of the 12 citrus COPT proteins; Table S1: Primers used in this study; Table S2: Identifiers and annotations of the PtCOPT and CsCOPT genes; Table S3: Inhibition-zone measurements from the plate-diffusion assay; Table S4: Nominal total Cu concentrations and Cu inputs of the tested formulations; Table S5: One-way ANOVA results and exact sample sizes for the data in Figure 4B, Figure 5A–F and Figure 6A–E; Table S6: Normalized expression values, with means, SE, n and *p* values, for the qRT-PCR data shown in Figure 7A.
