# Supplementary material for: Efficacy Evaluation of Different Copper-Based Bactericides Against Citrus Canker and Candidate Role of Citrus COPT Genes in Response to Xcc
Source: Plants (Basel). 2026 Nov 5;15(21):3453. doi: 10.3390/plants15213453 (PMC13765832; doi:10.3390/plants15213453)
Supplement: Supplementary file 1 [file plants-15-03453-s001.zip › plants-4556090-supplementary.pdf]

plants

MDPI

Supplementary Materials

Table S1. Primers used for real-time quantitative PCR in this study.

Primers	Sequences (5′–3′)
EF1α-F	GTAACCAAGTCTGCTGCCAAG
EF1α-R	GACCCAAACACCCAACACATT
qCOPT1-F	CTAGGCTTTGCTGTTGAAGTCTTGTCC
qCOPT1-R	CGACTATGAACACGCCAAGATTAAAGG
qCOPT2-F	TTCATGATCTCACTGACAGTGGAGTGG
qCOPT2-R	CATCACCAAATAGGCCAGTCCAATC
qCOPT3-F	GTGGCTGCTGGTTTGATTCAGACTC
qCOPT3-R	GGAGGAAGATCAGAGGTTTTTGGAGG
qCOPT4-F	ACGCCCTAGTCCTCTTCAAAGGTTG
qCOPT4-R	AGTGCAAGCATCACCAGATTGGC
qCOPT5-F	AAGGGCAGGCATGTATGCATTAGC
qCOPT5-R	AGCATCAGCAGATATCCAAGACTAACCC
qCOPT6-F	GTTCGACTTTTGGCGTACCGATTC
qCOPT6-R	GAATTGATCCCAAAGAGGGCCG

Table S2. Corresponding identifiers of six COPT genes in sweet orange and *Poncirus trifoliata*.

COPT member	Sweet orange gene ID	*Poncirus trifoliata* gene ID
COPT1	Cs6g07300	Ptrif.0006s0759.1
COPT2	Cs6g19470	Ptrif.0006s1990.1
COPT3	Cs8g19460	Ptrif.0008s2293.1
COPT4	Cs8g19450	Ptrif.0008s2292.1
COPT5	Cs8g19430	Ptrif.0008s2291.1
COPT6	Cs4g06330	Ptrif.0001s2061.1

Table S3. Inhibition-zone responses of commercial copper-containing formulations against *Xcc* in the plate-diffusion screening assay.

Code	Concentration / mg mL⁻¹	Inhibition-zone diameter / mm	Code	Concentration / mg mL⁻¹	Inhibition-zone diameter / mm
a	128	11.05	i	128	8.70
	64	7.56		64	6.02
	32	6*		32	6*
	16	6*		16	6*
	8	6*		8	6*
	0	6*		0	6*
b	128	12.9	j	128	8.10
	64	11.93		64	7.05
	32	10.71		32	6*
	16	9.50		16	6*
	8	6*		8	6*
	0	6*		0	6*
c	128	10.13	k	128	6*
	64	9.02		64	6*
	32	6.92		32	6*
	16	6*		16	6*
	8	6*		8	6*
	0	6*		0	6*
d	128	14.74	l	128	6*
	64	10.80		64	6*
	32	9.48		32	6*
	16	6.01		16	6*
	8	6*		8	6*
	0	6*		0	6*
e	128	12.76	m	128	21.13
	64	12.15		64	12.70
	32	9.36		32	10.28
	16	6.58		16	7.80
	8	6*		8	6*
	0	6*		0	6*
f	128	11.86	n	128	6*
	64	10.41		64	6*
	32	7.90		32	6*
	16	6*		16	6*
	8	6*		8	6*
	0	6*		0	6*

**Code**	**Concentration / mg mL⁻¹**	**Inhibition-zone diameter / mm**	**Code**	**Concentration / mg mL⁻¹**	**Inhibition-zone diameter / mm**
g	128	12.43	o	128	13.88
	64	10.85		64	9.53
	32	9.45		32	8.93
	16	7.64		16	6*
	8	6*		8	6*
	0	6*		0	6*
h	128	10.48	p	128	6*
	64	9.14		64	6*
	32	7.55		32	6*
	16	6*		16	6*
	8	6*		8	6*
	0	6*		0	6*

Formulation codes a–p correspond to the products listed in Table 1. A value of 6* indicates that no visible inhibition zone extended beyond the 6 mm filter-paper disc.

Table S4. Screening concentrations and nominal total Cu inputs in the root-drench assay.

Code	Nominal total Cu concentration (mM)	Nominal total Cu input per seedling (mg)	Nominal total Cu input per pot (mg)
a	0.4 / 4 / 12	2.542 / 25.418 / 76.255	10.167 / 101.674 / 305.021
b	0.1 / 1 / 4	0.635 / 6.355 / 25.418	2.542 / 25.418 / 101.674
c	0.5 / 5 / 15	3.177 / 31.773 / 95.319	12.709 / 127.092 / 381.276
d	0.1 / 1 / 3	0.635 / 6.355 / 19.064	2.542 / 25.418 / 76.255
e	0.4 / 4 / 12	2.542 / 25.418 / 76.255	10.167 / 101.674 / 305.021
f	0.1 / 1 / 5	0.635 / 6.355 / 31.773	2.542 / 25.418 / 127.092
g	0.1 / 1 / 5	0.635 / 6.355 / 31.773	2.542 / 25.418 / 127.092
h	0.1 / 1 / 5	0.635 / 6.355 / 31.773	2.542 / 25.418 / 127.092
i	0.1 / 1 / 3	0.635 / 6.355 / 19.064	2.542 / 25.418 / 76.255
j	0.1 / 1 / 3	0.635 / 6.355 / 19.064	2.542 / 25.418 / 76.255
k	0.1 / 1 / 4	0.635 / 6.355 / 25.418	2.542 / 25.418 / 101.674
l	0.1 / 1 / 4	0.635 / 6.355 / 25.418	2.542 / 25.418 / 101.674
m	0.1 / 1 / 4	0.635 / 6.355 / 25.418	2.542 / 25.418 / 101.674
n	0.1 / 1 / 4	0.635 / 6.355 / 25.418	2.542 / 25.418 / 101.674
o	0.1 / 1 / 5	0.635 / 6.355 / 31.773	2.542 / 25.418 / 127.092
p	0.1 / 1 / 4	0.635 / 6.355 / 25.418	2.542 / 25.418 / 101.674

Codes a–p correspond to Table 1. Concentrations were converted from label-recommended field rates to nominal total Cu; the product-specific values are listed above. At 100 mL per seedling and four seedlings per pot, the nominal total Cu input per application is C × 6.3546 mg per seedling or C × 25.4184 mg per pot, where C is the nominal total Cu concentration in mM. Inputs are calculated from nominal concentrations and application volumes for a single application. The water control contains no added Cu.

Table S5. One-way ANOVA results, assumption tests, post hoc comparisons, and exact sample sizes for Figures 4B, 5A–F, and 6A–E. (Excel file)

Table S6. Replicate-level qPCR values, means ± SE, one-way ANOVA, and Dunnett comparisons for Figure 7A. (Excel file)

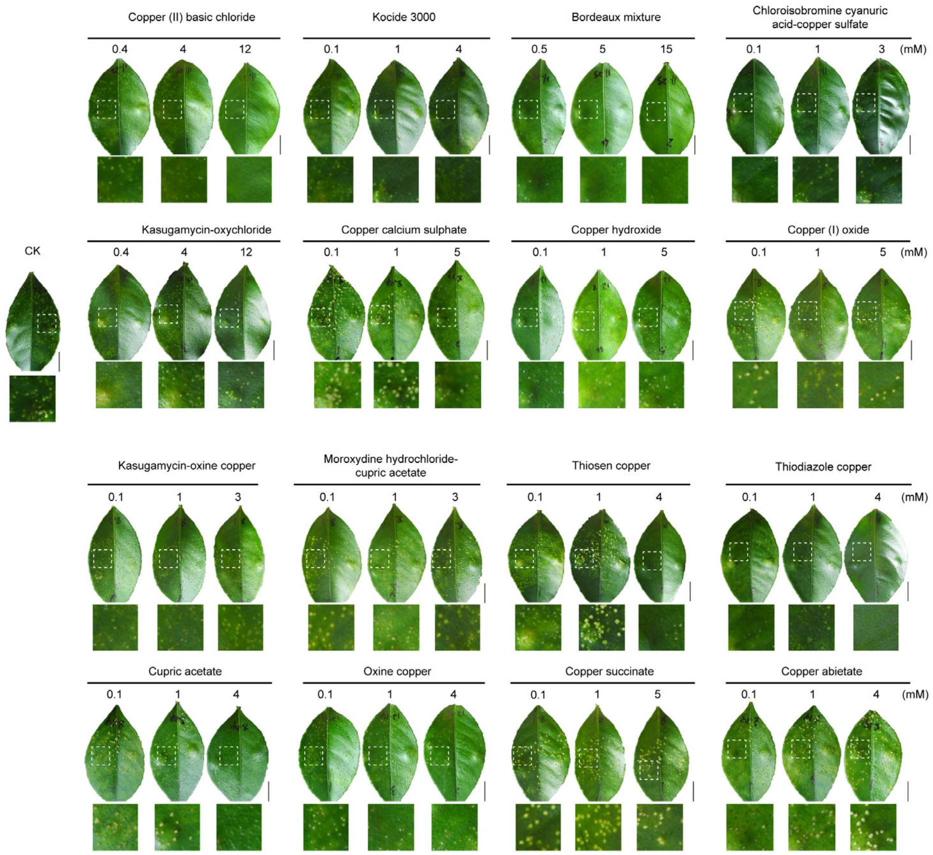

Figure S1. Citrus canker symptoms on the adaxial surface of sweet orange leaves.

Plants were treated by root drenching with the indicated commercial copper-containing formulations for 14 days before *Xcc* inoculation. Treatment levels are expressed as nominal total Cu concentrations (mM). Leaves were infiltrated with *Xanthomonas citri* subsp. *citri* (*Xcc*) at 10^4 CFU mL^{-1}. Photographs show disease symptoms on the adaxial leaf surface at 14 days post-inoculation (dpi). The scale bar represents 1 cm.

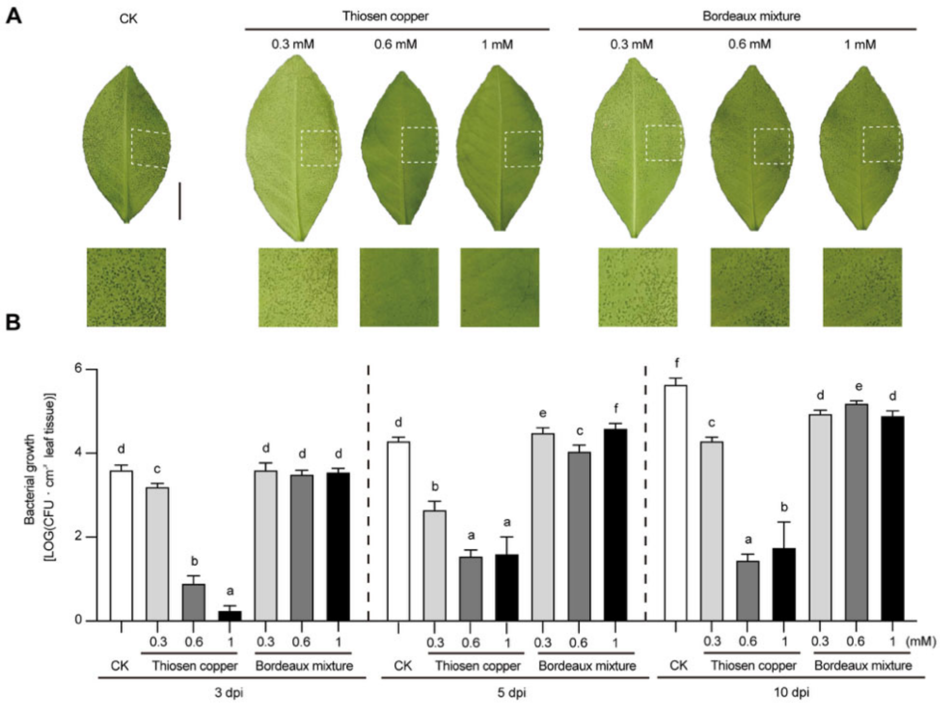

Figure S2. Effects of Thiosen Copper and Bordeaux Mixture at low concentrations on citrus canker symptoms and bacterial populations.

A, Symptoms of citrus canker on sweet orange leaves treated with Thiosen Copper or Bordeaux Mixture. Dashed boxes indicate magnified lesion views. The scale bar represents 1 cm. B, Bacterial populations in leaf tissues at 3, 5, and 10 days post-inoculation (dpi). Sweet orange seedlings were treated by root drenching for 14 days before inoculation with *Xanthomonas citri* subsp. *citri* (*Xcc*; 10^4 CFU mL^{-1}). Treatment levels are expressed as nominal total Cu concentrations of 0.3, 0.6, and 1 mM. CK represents the water control. Data are means ± SE (n = 3 pots). Each sampling time was analyzed separately by one-way ANOVA followed by Duncan's new multiple range test; different letters indicate significant differences at $P < 0.05$.

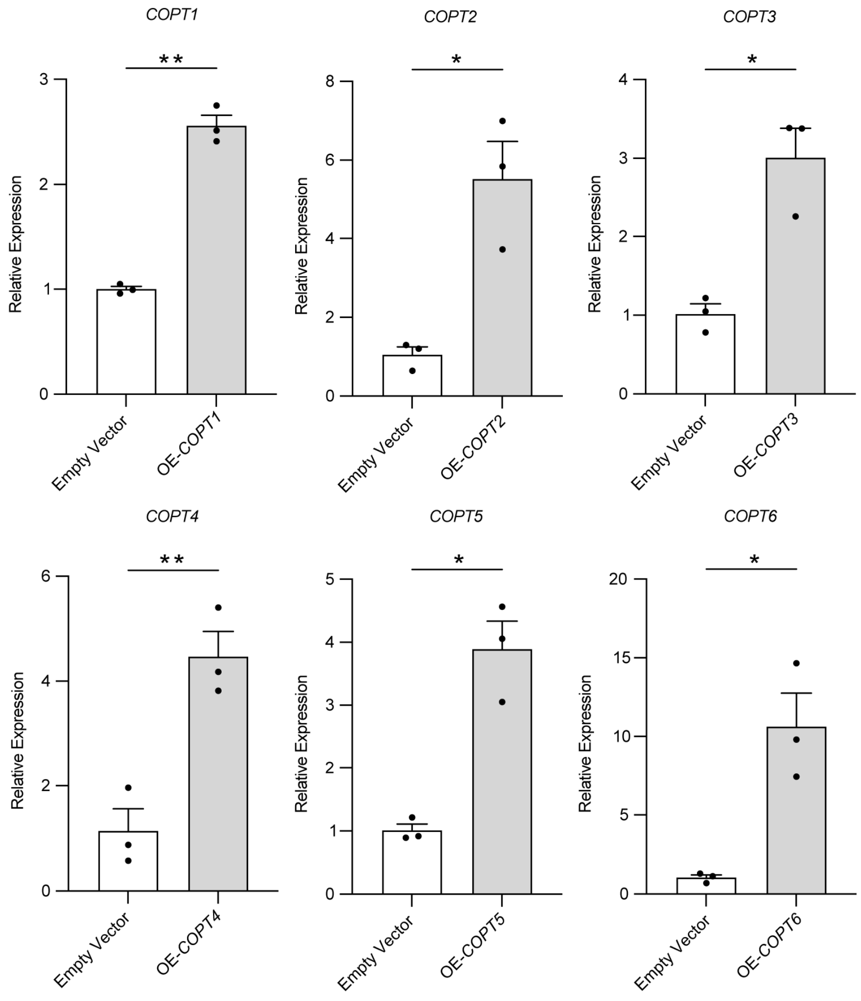

Figure S3. Validation of transient *CsCOPT1–CsCOPT6* overexpression in sweet orange leaves by qRT-PCR.

Relative expression levels of *CsCOPT1*, *CsCOPT2*, *CsCOPT3*, *CsCOPT4*, *CsCOPT5*, and *CsCOPT6* in sweet orange leaves transiently transformed with the corresponding overexpression constructs or the empty vector control. Transcript levels were determined by qRT-PCR in tissues sampled at 2 dpi to confirm increased target-gene transcript abundance. Data represent means ± SE (n = 3 independent plants per group). A single asterisk (*) indicates a significant difference at $P < 0.05$, and a double asterisk (**) indicates a significant difference at $P < 0.01$, as determined by a two-tailed Welch's unpaired t-test on ΔCt; each panel compares one construct with its matched EV, with no multiple-comparison correction across genes.

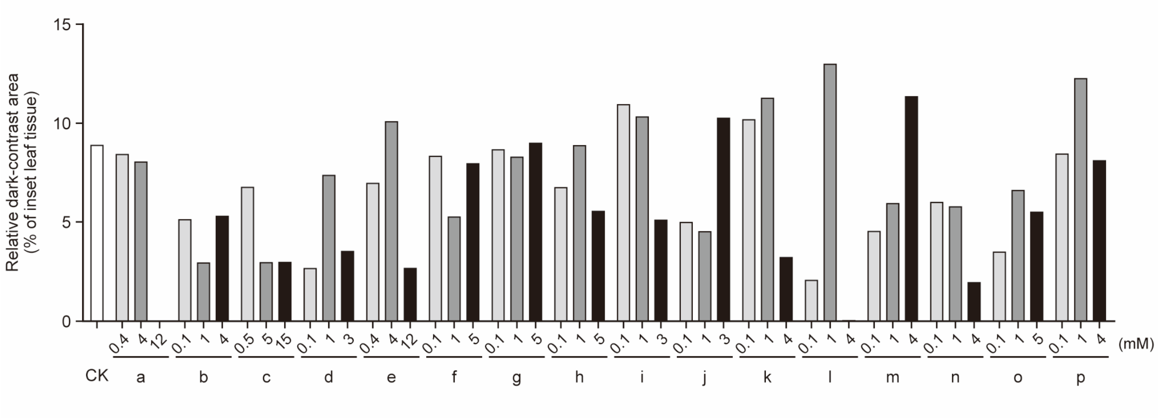

Figure S4. Estimated lesion area in sweet orange leaf insets from the commercial-formulation screening assay.

Lesion-area estimates for 49 displayed leaf insets from Figure 2 at 14 dpi: one water control and three treatment levels for each of 16 formulations. Values are the percentage of inset leaf-tissue area assigned to lesions by image segmentation, as defined in Section 2.10. Codes a–p correspond to Table 1 and Figure 2. White denotes CK; light gray, dark gray, and black indicate increasing nominal total Cu concentrations within each formulation. Organic/inorganic labels denote formulation groups. Each bar represents one displayed region. A zero value indicates that no lesion area was detected at the selected threshold.

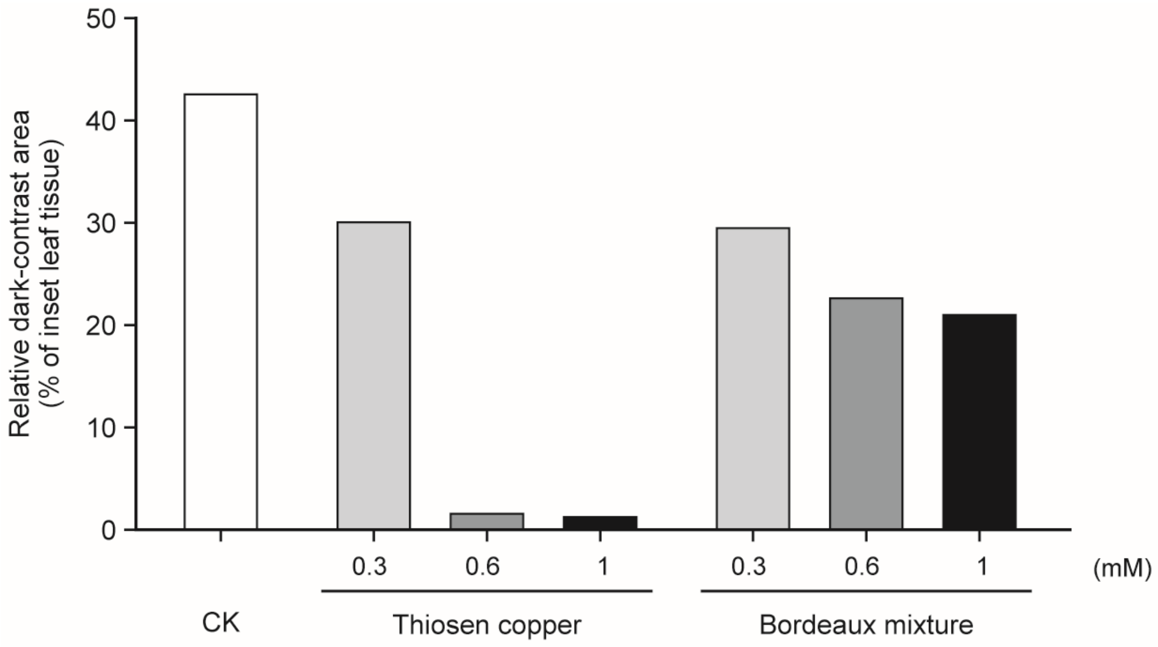

Figure S5. Estimated lesion area in sweet orange leaf insets following Thiosen Copper or Bordeaux Mixture treatment.

Lesion-area estimates for seven displayed leaf insets from Figure S2A: CK and three treatment levels for each formulation. Values were calculated as described in Section 2.10. White denotes CK; light gray, dark gray, and black denote nominal total Cu concentrations of 0.3, 0.6, and 1 mM, respectively. Each bar represents one displayed region.

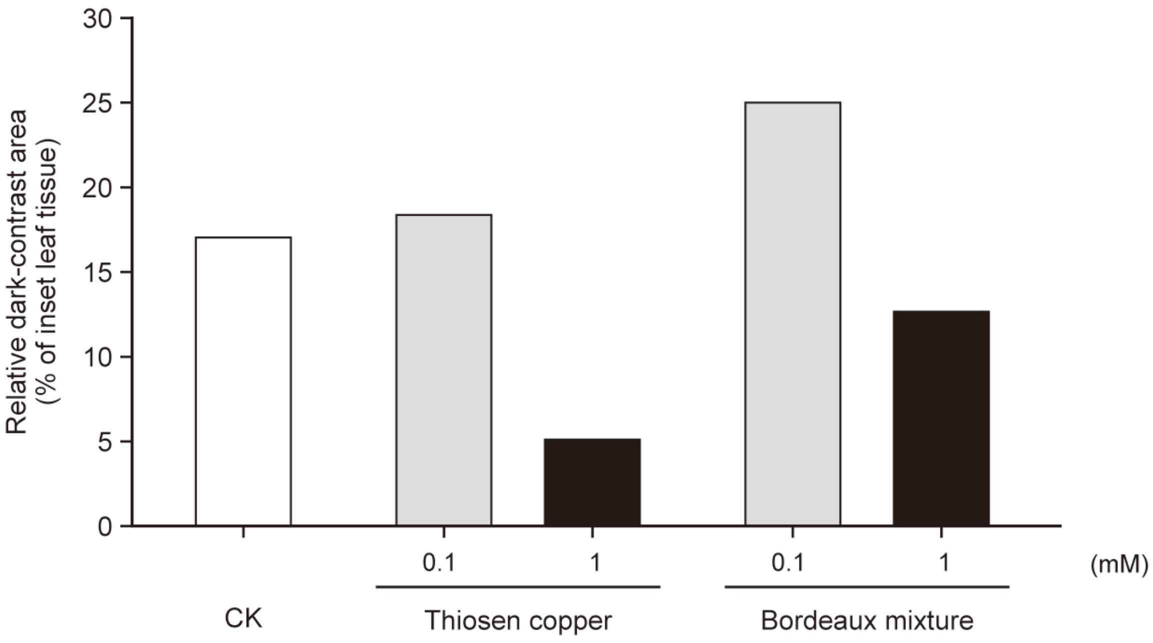

Figure S6. Estimated lesion area in *Poncirus trifoliata* leaf insets following Thiosen Copper or Bordeaux Mixture treatment.

Lesion-area estimates for five displayed leaf insets from Figure 4A at 14 dpi: CK and two treatment levels for each formulation. Values were calculated as described in Section 2.10. White denotes CK; light gray and black denote nominal total Cu concentrations of 0.1 and 1 mM, respectively. Each bar represents one displayed region.

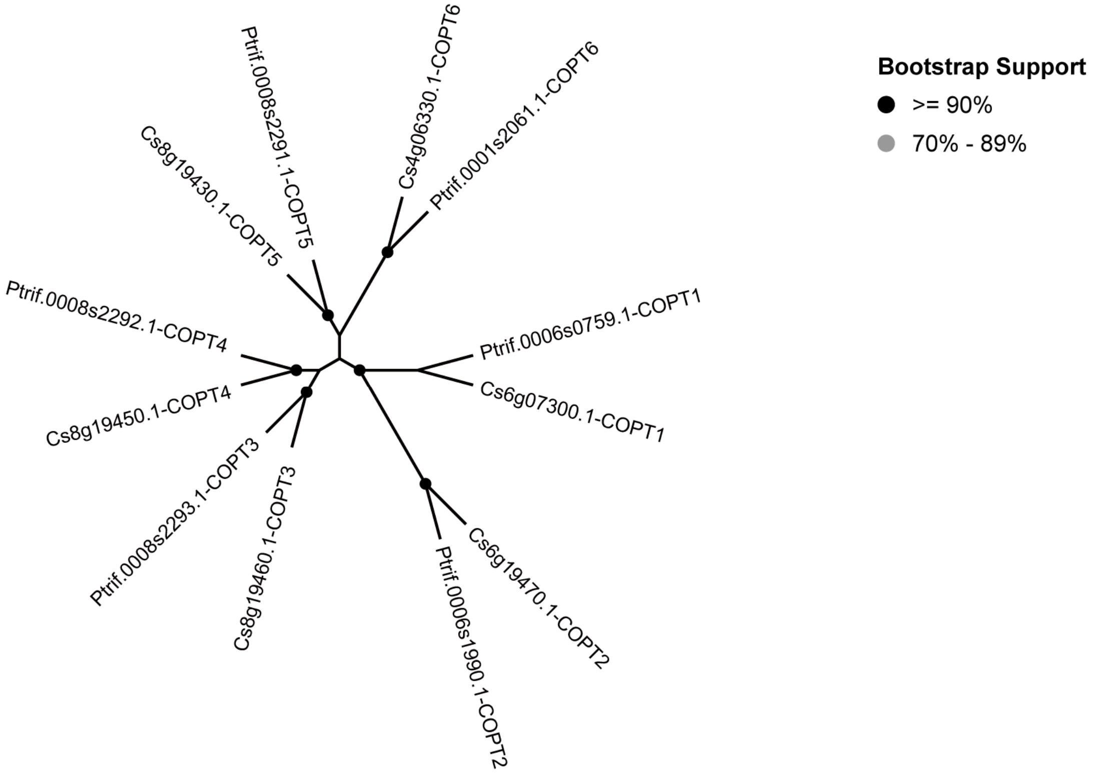

Figure S7. Unrooted maximum-likelihood phylogenetic tree of citrus COPT proteins.

The tree includes 12 protein sequences: *PtCOPT1–PtCOPT6* and *CsCOPT1–CsCOPT6* (667 amino-acid sites). The best-fit model was VT+G4; the tree was inferred in IQ-TREE 3.0.1 with 1000 ultrafast bootstrap replicates (seed 991264). Node symbols indicate ultrafast bootstrap support: black, ≥90%; grey, 70–89%; values below 70% are not shown. The tree is unrooted, and no outgroup was included. Five Pt/Cs pairs (COPT2–COPT6) formed supported sister pairs; COPT1 did not form a supported pair, and its correspondence remains annotation-based

https://doi.org/10.3390/xxxxx
